# Plerixafor and granulocyte colony stimulating factor for poor mobilizers in patients undergoing autologous peripheral hematopoietic stem cell transplantation: Single institution study

**DOI:** 10.3389/frtra.2022.1017579

**Published:** 2023-01-18

**Authors:** Jean El Cheikh, Khodr Terro, Samantha El Warrak, Nohra Ghaoui, Layal Sharrouf, Michael Anthony Timonian, Fatima Ismail, Ammar Zahreddine, Nabila Kreidieh, Nour Moukalled, Iman Abou Dalle, Ali Bazarbachi

**Affiliations:** ^1^ Division of Hematology/Oncology, Department of Internal Medicine, American University of Beirut Medical Center, Beirut, Lebanon; ^2^ Bone Marrow Transplantation Program, Department of Internal Medicine, American University of Beirut Medical Center, Beirut, Lebanon; ^3^ Faculty of Medicine, American University of Beirut, Beirut, Lebanon; ^4^ Department of Pharmacy, American University of Beirut Medical Center, Beirut, Lebanon; ^5^ Nursing Administration, American University of Beirut Medical Center, Beirut, Lebanon; ^6^ Stem Cell Processing Laboratory, Department of Pathology and Laboratory Medicine, American University of Beirut Medical Center, Beirut, Lebanon

**Keywords:** plerixafor, granulocyte colony stimulating factor, poor mobilizers, autologous hematopoietic stem cell transplantation plerixafor for stem cell mobilization and transplantation, plerixafor (Mozobil), autologous transplant, myeloma, lymphoma

## Abstract

**Background:**

Autologous hematopoietic stem cell transplantation (ASCT) has become the mainstay treatment for many hematological malignancies and solid tumors. An adequate number of stem cells must be collected for better ASCT outcomes, which is challenging in 5%–30% of patients. To improve mobilization, plerixafor is used along with granulocyte colony-stimulating factor (G-CSF).

**Patients and methods:**

We conducted a retrospective single center study involving patients who received plerixafor pre-ASCTs between January 2013 and December 2020 at a tertiary care center in Lebanon. We identified a total of 84 consecutive adult patients. All patients identified were poor mobilizers and have eventually received plerixafor either as pre-emptive use before first apheresis in those with peripheral CD34 + of less than 20 cells/ul, or after failure of first apheresis in those with peripheral stem cells (PSC) >2.0 × 10^6^ cells/Kg.

**Results:**

The median age at ASCT was 52.7 years (22–74) with 61% male predominance. Multiple myeloma was the most prevalent disease 64% followed by Lymphoma 32%. The majority of patients were in complete remission 64% at the time of ASCT. Most patients received proteasome inhibitor-based induction therapy 67% and Melphalan-based conditioning therapy 68%. The median follow-up from ASCT was 9 months (1–59). It was noted that greater body mass index (BMI) is a significant factor for better PSC collection whether premobilization (*P* = 0.003), or post plerixafor mobilization (*P* = 0.024). Moreover, Multiple Myeloma patients showed better mobilization using Plerixafor (*P* = 0.049). Using Plerixafor along with G-CSF in poor mobilizers post G-CSF alone showed a statistically significant increase in the collected PSC mean from 0.67 × 10^6^ cells/Kg to 4.90 × 10^6^ cells/Kg (*P* < 0.001) with a failure rate only for 12 patients (15%). The infusion of PSC > 2.5 × 10^6^ cells/Kg has shown 3 days decrease in time to platelet engraftment (*P* = 0.021) and a 36% decrease in progression/relapse rate (*P* = 0.025).

**Conclusion:**

Plerixafor is effective in increasing the PSC yield in poor mobilizers. Low BMI and hematologic malignancies other than Multiple Myeloma are risk factors for poor mobilization. More studies should be performed to establish more risk factors, helping us to identify poor mobilizers more accurately and initiate plerixafor mobilization early on.

## Introduction

High-dose chemotherapy is the standard of treatment for a wide array of hematological malignancies and solid tumors prior to autologous hematopoietic stem cell transplantation (ASCT) (1). ASCT is the treatment of choice for relapse and refractory transplant-eligible cases as it leads to improved progression-free survival and overall survival (2). Cytotoxic chemotherapy, used initially for treatment of primary disease, poses extensive damage on the bone marrow, and therefore it is vital to collect an adequate number of peripheral blood stem cells (PBSCs) to end up with a successful transplantation. A successful hematopoietic stem cell transplant (HSCT) depends largely on an optimal number of CD34 + cells in the peripheral blood that can be harvested (2). The American Society for blood and marrow transplantation recommends 4–5 × 10^6^ CD34 + cells/kg as the optimal number of hematopoietic stem cells needed to undergo a successful transplant and ≥2 × 10^6^ CD34 + cells/kg as the minimal number required (2).The recommended optimal number of CD34+ is associated with faster neutrophil and platelet recovery, reduced hospitalization, blood transfusions, and antibiotic therapy (3). Infusion of CD34 + cell doses <1.5–2.5 × 10^6^/kg leads to delayed neutrophil and platelet recovery. There are many cases however, that cannot mobilize or may require multiple attempts in order to perform a successful HSCT. Those cases are called poor mobilizers and they range between 5% and 30% (4).

The general approach towards stem cell collection involves cytokine mobilization using granulocyte-colony stimulating factor (G-CSF) or granulocyte macrophage-colony stimulating factor (GM-CSF) alone or in combination, and chemo-mobilization (CM) using chemotherapy followed by administration of growth factors (5). G-CSF is the most potent of the myeloid growth factors and works by inducing the release of various proteases into the marrow, which then cleaves adhesion molecules such as Stromal Cell Derived Factor (SDF-1), releasing hematopoietic stem cells into the peripheral blood (6). Chemotherapy, high dose cyclophosphamide with or without other agents enhances CD34 + mobilization (7). Chemotherapy-based mobilization is widely used and represents standard of care in some transplant institutions (7). Chemotherapy mobilizes HSCs to the peripheral blood by compensatory neutrophil production following chemotherapy-induced aplasia (3). Other regimens used for stem cell mobilization include ICE (Ifosfamide, carboplatin, etoposide), RICE (rituximab + ICE), DHAP (cisplatin, cytarabine, dexamethasone) etc (8, 9). The drawbacks of using chemotherapy for mobilization is related to the toxicities and complications associated with their use (10). Today, 10%–25% of patients fail to obtain sufficient CD34 + cell yields to proceed to ASCT with the standard G-CSF/chemotherapy regimen (3).

In December 2008, the Food and Drug Administration approved the use of plerixafor in combination with G-CSF to mobilize hematopoietic stem cells to the peripheral blood pre-ASCT (10). Plerixafor in combination with G-CSF has shown to significantly increase the number of peripheral CD34 + cells as compared to G-CSF alone when used upfront in multiple myeloma (MM), non-Hodgkin lymphoma (NHL), and Hodgkin lymphoma (HL) patients undergoing ASCT (3). Plerixafor induces the mobilization of stem cells into the bloodstream by reversibly binding to chemokine receptor CXCR4 and antagonizing the chemokine stromal cell-derived factor-1α (SDF-1α) interaction (4). The addition of plerixafor to G-CSF was found to significantly reduce the mobilization failure rates from 75% to 27% (11). It has been implemented pre-ASCT for patients in whom initial mobilization was either predicted to fail or has already failed (12). A number of factors are known to negatively affect the outcome of stem cell mobilization and these include but are not limited to advanced age, a diagnosis of NHL, previous radiotherapy, number of lines of chemotherapy pre-ASCT, exposure to lenalidomide or purine analogues and failure of prior mobilization (12). Increased circulating tumor cells have been reported in acute myeloid leukemia and plasma cell leukemia patients. Therefore, plerixafor is not recommended for use in leukemia patients (3).

When plerixafor is combined with G-CSF, hematopoietic stem cell mobilization is enhanced compared with either plerixafor or G-CSF alone. CD34 + cell counts usually peak 10–14 h following administration of plerixafor (3).

## Methods

### Patient population and data collection

We conducted a retrospective single-center study involving patients aged ≥18 years who received plerixafor pre-ASCT between January 2013 and January 2021 at the American University of Beirut Medical Center (AUBMC), a tertiary care center in Lebanon. We identified a total of 84 consecutive adult patients who were administered plerixafor for peripheral stem cell mobilization. Patients who received plerixafor were divided into poor mobilizers with pre-emptive use before first apheresis in those with peripheral CD34 + stem cells (PSC) <20 cells/uL, or after failure of first apheresis in those with PSC ≥ 20 cells/uL. The following data were extracted: Date of birth, gender, body mass index, ABO blood group, date of diagnosis, age at diagnosis, disease type, bone marrow involvement, lines of chemotherapy, CD34 + cells pre-plerixafor (cell/uL), collected peripheral CD34 + cells pre-plerixafor (×10^6^/kg), number of plerixafor vials and doses used, number of apheresis attempts, total collected CD34 + cells post-plerixafor (×10^6^/kg), dose of dimethylsufoxide (DMSO), collection date, number of platelets pre-ASCT, conditioning regimen, date of transplant, disease status at transplant, length of hospital stay, date to Absolute Neutrophil Count (ANC) and platelet engraftment, development of central line blood stream infection (CLABSI) and clostridium *difficile* infections, and date of disease relapse, disease progression, or death.

### Interventions

The drug was used in accordance with registration guidelines of plerixafor. The protocol used for mobilization includes a daily subcutaneous injection of G-CSF (10 µg/Kg), for four consecutive days before administering plerixafor to the patient. On the evening of the fourth day, patients received a subcutaneous injection of plerixafor (240 µg/Kg) 10–12 h before collecting stem cells. In our study, all patients received 240 µg/kg Plerixafor plus 10 µg/kg G-CSF prior to collection. Patients should have vascular access evaluated before the start of any apheresis procedure. Optimal mobilization requires the collection of the targeted stem cell dose, strategies to minimize the number of apheresis sessions required, cost reduction, and avoiding mobilization-related complications, such as hospitalization for febrile neutropenia (2). At AUBMC, plerixafor is available in single-use vials containing 1.2 ml of 20 mg/ml solution containing 24 mg of plerixafor for subcutaneous injection. It is used in combination with G-CSF to improve collection for autologous stem cell transplantation. The standard operations procedure at our institution allows plerixafor administration when peripheral CD34 + cell count is <20 cells/µl when G-CSF is used alone as mobilization strategy. The dose of plerixafor used is 240 µg/kg actual body weight, which can be safely increased to a maximum dose of 24 mg (accounting for a full vial) adjusted for kidney function as appropriate. The patient can be re-dosed if a second apheresis is needed. The mean peak time of plerixafor is 11 h (10–14 h). Apheresis is started around 8 h after administration of plerixafor to allow collection during the peak. All collection procedures were done by Large Volume Leukapheresis (LVL) and Continuous Flow Mononuclear Cell (CMNC) collection technique using Cobe-Spectra and Optia apheresis machines.

The time difference between two apheresis procedures, with or without plerixafor is approximately 24 h (minimum of 20 h). The time needed for plerixafor to mobilize hemoprogenitor cells from the bone marrow niche to the peripheral blood is about 8–11 h (13). Thus, plerixafor is usually given at midnight and apheresis take places at around 8:00 am. Apheresis usually takes around 3–4 h until an optimal collect is obtained. Engraftment date is the first day of three consecutive days where ANC > 500 cells/ml with or without G-CSF support, regardless of what the numbers are on subsequent days. Platelet engraftment is on the first of seven days where platelet count sustains >20,000/ml, a week away from any platelet transfusion (14).

Moreover, all our patients had disease evaluation after 3–4 cycles of treatment for multiple myeloma including bone marrow biopsy and full multiple myeloma work up as recommended by the international myeloma working group, for diagnosis and disease evaluation (15).

### Primary and secondary endpoints

The primary endpoint of the study is to evaluate the real-life efficacy of plerixafor and to collect ≥2.5 × 10^6^ CD34 + cells/kg optimal to proceed to ASCT. Secondary endpoints include identifying factors associated with poor mobilization, stem cell yield pre- and post-plerixafor as well as time to neutrophil and platelet engraftment post ASCT.

### Ethical considerations

The Institutional Review Board at the American University of Beirut approved the study after taking into consideration all the safety measures taken to preserve patients' confidentiality. The study does not involve any diagnostic or therapeutic intervention and there was no risk of harm to the patients.

## Statistical analysis

Patients' characteristics were summarized using descriptive statistics, including median (range) for continuous variables and frequency (percentage) for categorical variables. The *χ*^2^ test was used to compare categorical variables. Fisher's exact test was considered to evaluate significance instead of Pearson's *χ*^2^ in small group stratifications. The survival rates were estimated by the Kaplan–Meier method and compared by a log-rank test. All *P-*values were 2-sided, with a significance level of .05. Multivariate analysis for CD 34 + mobilization was performed using a binary logistic regression with using backward stepwise elimination process in which only significant variables are kept at the last step. All statistical analyses were performed using IBM SPSS Statistics, version 27.

## Results

### Patient characteristics

We identified 84 adult patients who had received plerixafor and underwent autologous hematopoietic stem cell transplantation at AUBMC. 61% of the patients were males. Median age at ASCT was 52.7 years (22.0–74.0). Median body mass index was 26.8 (17.0–39.0). Multiple myeloma was the most predominant disease found amongst our study population accounting for 64% of the patients. 13% of patients had NHL, and 9% of patients had HL. 3% of patients had other oncologic conditions consisting of one patient with testicular cancer, the second with Ewing sarcoma and the third patient with Waldenstrom's macroglobulinemia. 64% of patients were in complete remission (CR) pre-ASCT. 70% of patients had bone marrow involvement with 13% comprising fluorescence in-situ hybridization (FISH) abnormalities. Prior to ASCT, 51% of patients had received at least 1 line of chemotherapy, and 33% had received >3 lines. Bortezomib was amongst the most common induction regimen administered to the patients [55% with some immunomodulatory agents (Imid) therapy and 12% without Imid therapy], possibly attributed to the fact that MM was the most common disease in our study population. All MM and Waldenstrom's macroglobulinemia patients received either PI alone based chemotherapy, Imid alone based chemotherapy or both combined. However, lymphoma patients have received intensive chemotherapy regimens.

Amongst all diseases, only 18% of patients had total collected CD34 + cells >2.5 × 10^6^ cells/kg post G-CSF and pre-plerixafor. This number increased to 84% of patients with total collected CD34 + cells >2.5 × 10^6^ cells/kg post G-CSF and plerixafor. 80% patients had low PSCs, requiring pre-emptive plerixafor prior to apheresis. 22% of patients were chemo-mobilized with DHAP (with or without rituximab (DHAP, R-DHAP) (Rituximab + Dexamethasone + Cytarabine + Cisplatin), R-ICE (Rituximab + Ifosfamide + Carboplatin + Etoposide) in conjunction with plerixafor. 23% of the patients have undergone preemptive apheresis. The conditioning regimen used was predominantly single-agent melphalan 73% and that is also attributed to the fact that MM is the dominant disease in our study. All our patients with lymphoma (HL or NHL) received BEAM conditioning. 2 of our patients with solid tumors have received thiotepa, busulfan, and +cyclophosphamide or carboplatin and etoposide as conditioning regimen.

Median follow up is 14 months (9–50). The median length of hospital stay for ASCT is 21 days (14–69). The median time needed for ANC and platelet engraftment are 11 days (4–28) and 18 days (4–48) respectively. The 1-year PFS and OS rates are 81% and 94% respectively. During the period of hospitalization, in the first 30 days, the mortality incidence is 1 patient (1%) who died from disease progression. At 100 days one more patient died also due to progression of disease marking the incidence of death as 2% by then. However, the incidence of death at 1 year reached 6% (5 patients) where 3 additional patients have died, 2 of which due to infection and the third due to hemorrhage. Only 7% suffered from central line associated blood stream infection Table 1.

**Table 1:** Patient characteristics.

	*N* (%)^a^ or median (range)
Number of Patients Included in Study	84
Gender	
Male	51 (61%)
Female	33 (39%)
Median Age at Auto-SCT	52.7 (22.0–74.0)
Blood Type	
A	37 (44%)
O	26 (31%)
B	15 (18%)
AB	6 (7%)
Body Mass Index (BMI)	26.8 (17.0–39.0)
Disease	
Multiple Myeloma	54 (64%)
Lymphoma	27 (32%)
Other^b^	3 (4%)
Disease Status at Transplant^c^	
CR	54 (64%)
PR/SD	27 (32%)
PD	3 (1%)
FISH abnormality	11 (13%)
* (MM patients with Del 13q, Del 17p, Trisomy 17, t (11,14), t (4,14), Del 4)*	
Bone marrow involvement	59 (70%)
Lines before Auto SCT	
1	43 (51%)
2	14 (17%)
3	13 (16%)
4 or more	14 (17%)
Induction^d^	
PI based	56 (67%)
Bortezomib with Imid	46 (55%)
Bortezomib without Imid	10 (12%)
Intensive Chemotherapy	21 (25%)
Imid based	7 (8%)
Peripheral CD34+ (×10^6^ cells/Kg) pre-Plerixafor	
<2.5	67 (80%)
>2.5	16 (20%)
Number of Plerixafor Vials Used	
1	59 (70%)
>1	25 (30%)
Collected CD34 (×10^6^ cells/Kg) post-GCSF^e^ & Plerixafor	
≤2.5	13 (16%)
>2.5	70 (84%)
Infused CD34 (×10^6^ cells/Kg)	
≤2.5	8 (10%)
>2.5	72 (90%)
Patients undergone preemptive apheresis	19 (23%)
Patients Chemo-Mobilized	18 (22%)
Conditioning Regimen	
Melphalan based	61 (73%)
BEAM^f^	21 (25%)
Other^g^	2 (2%)
Median follow up in months (range)	14 (9–50)
Length of inpatient stay during Auto SCT in days, mean (range)	20.9 (14–69)
Days to ANC engraftment, mean (range)	11.3 (4–28)
Days to Platelet engraftment, mean (range)	17.9 (4–48)
1 year PFS	68 (81%)
Overall Survival Status	
Dead	12 (14%)
Disease	7 (8%)
TRM	5 (6%)
Alive	72 (86%)
Incidence of death at 30 days	1 (1%)
Cause of death	
Disease	1 (1%)
TRM	0 (0%)
Incidence of death at 100 days	2 (2%)
Cause of death	
Disease	2 (2%)
TRM (transplant-related mortality)	0 (0%)
Incidence of death at 1 year	5 (6%)
Cause of death	
Disease	2 (2%)
TRM	3 (4%)
1 year OS	79 (94%)
CLABSI^h^	
Yes	6 (7%)
No	75 (93%)

^a^ Percentages may not add up to 100% due to rounding.

^b^ Includes two solid tumors and one Waldenstrom's Macroglobulinemia.

^c^ CR, complete remission; PD, progression of disease; PR, partial response dIMID, immunomodulatory; PI, proteasome inhibitor.

^d^ IMID, immunomodulatory; PI, proteasome inhibitor.

^e^ Granulocyte colony-stimulating factor.

^f^ BEAM, carmustine, etoposide, aracytin and melphalan.

^g^ Includes: Thiotepa + Busulfan + Cyclophosphamide or Carboplatin + Etoposide.

^h^ CLABSI, central line associated blood stream infection.

On Univariate and Multivariate analysis of pre-ASCT variables in association with total collected cells CD34 + pre plerixafor and post G-CSF and plerixafor, it is noted that patient BMI, BM involvement and disease type are the only significant variables. MM and greater BMI showed a statistically significant greater amount of CD34 + count pre-plerixafor with odds ratios (OR) of 4.349 and 1.215 and *P*-values of 0.027 and 0.009 respectively. On multivariate analysis BMI was the only variable that showed a statistically significant positive correlation with collected CD34 + post-GCSF and plerixafor (OR = 1.254) and (*P* = 0.018) Tables 2, 3.

**Table 2:** Univariate analysis of variables in association with mobilization outcomes.

		Peripheral CD34+ (pre-plerixafor)	*P*-value	Collected CD34 (post-GCSF^a^ & plerixafor)	Collected CD34 (post-GCSF^a^ & plerixafor)	*P*-value
Variable	≤2.5 (×10^6^ cells/Kg)	>2.5 (×10^6^ cells/Kg)	–	≤2.5 (×10^6^ cells/Kg)	>2.5 (×10^6^ cells/Kg)	–
*N* (%)^b^ or mean	(*N* = 67)	(*N* = 16)		(*N* = 13)	(*N* = 70)	
Gender			0.439			0.754
Male	39 (58%)	11 (69%)		12 (92%)	38(62%)	
Female	28 (42%)	5 (31%)		1 (8%)	32 (38%)	
Age at Auto-SCT mean (range)	53.1 (22.0–74.0)	53.0 (31.0–63.0)	0.989	52.6 (22.0–70.0)	53.5 (23.0–74.0)	0.746
BMI mean (range)	26.2 (17.0–38.7)	39.7 (22.7–39.0)	0.003	25.9 (17.0–39.0)	28.2 (20.0–38.7)	0.024
Disease			0.027			0.049
MM	39 (58%)	15 (94%)		8 (62%)	46 (66%)	
Lymphoma	25 (37%)	1 (0%)		5 (38%)	21 (30%)	
Other^c^	3 (5%)	0 (0%)		0 (0%)	3 (4%)	
BM^d^ involvement	44 (68%)	15 (94%)	0.036	8 (62%)	51 (75%)	0.317
Lines before Auto SCT			0.842			0.158
1	33 (50%)	10 (62%)		6 (50%)	37 (53%)	
2	12 (18%)	2 (13%)		2 (17%)	12 (17%)	
3	11 (17%)	2 (13%)		1 (8%)	12 (17%)	
4 or more	10 (15%)	2 (13%)		3 (25%)	9 (13%)	
Status at Transplant^e^			0.553			0.490
CR	27 (44%)	9 (56%)		14 (39%)	22 (52%)	
PR/SD	33 (53%)	7 (44%)		21 (58%)	19 (45%)	
PD	2 (3%)	0 (0%)		1 (3%)	1 (2%)	
Induction^f^			0.742			0.327
PI based						
Bortezomib with Imid	37 (55%)	9 (56%)		7 (56%)	39 (56%)	
Bortezomib without Imid	7 (10%)	3 (19%)		2 (10%)	8 (11%)	
Intensive Chemotherapy	20 (30%)	1 (6%)		3 (26%)	18 (26%)	
Imid based	3 (5%)	3 (19%)		1 (7%)	5 (7%)	

^a^ Granulocyte colony-stimulating factor.

^b^ Percentages may not add up to 100% due to rounding.

^c^ Includes two solid tumors and one Waldenstrom's Macroglobulinemia.

^d^ Bone Marrow.

^e^ CR, complete remission; PR, progression of disease; SD, stable disease; PR, partial response.

^f^ PI, proteasome inhibitor; IMID, immunomodulatory.

**Table 3:** Multivariate analysis of variables in association with mobilization outcomes.

	OR	95% CI	*P*-Value
BMI mean (pre-plerixafor)	1.215	1.049–1.406	0.009
Disease MM vs. others (pre-plerixafor)	4.349	0.494–38.315	0.027
BM involvement (pre-plerixafor)	1.241	0.038–40.408	0.080
BMI mean (post-GCSF & plerixafor)	1.254	1.040–1.512	0.018
Disease MM vs. others ((post-GCSF & plerixafor)	0.738	0.154–3.548	0.359

**Figure 1:**
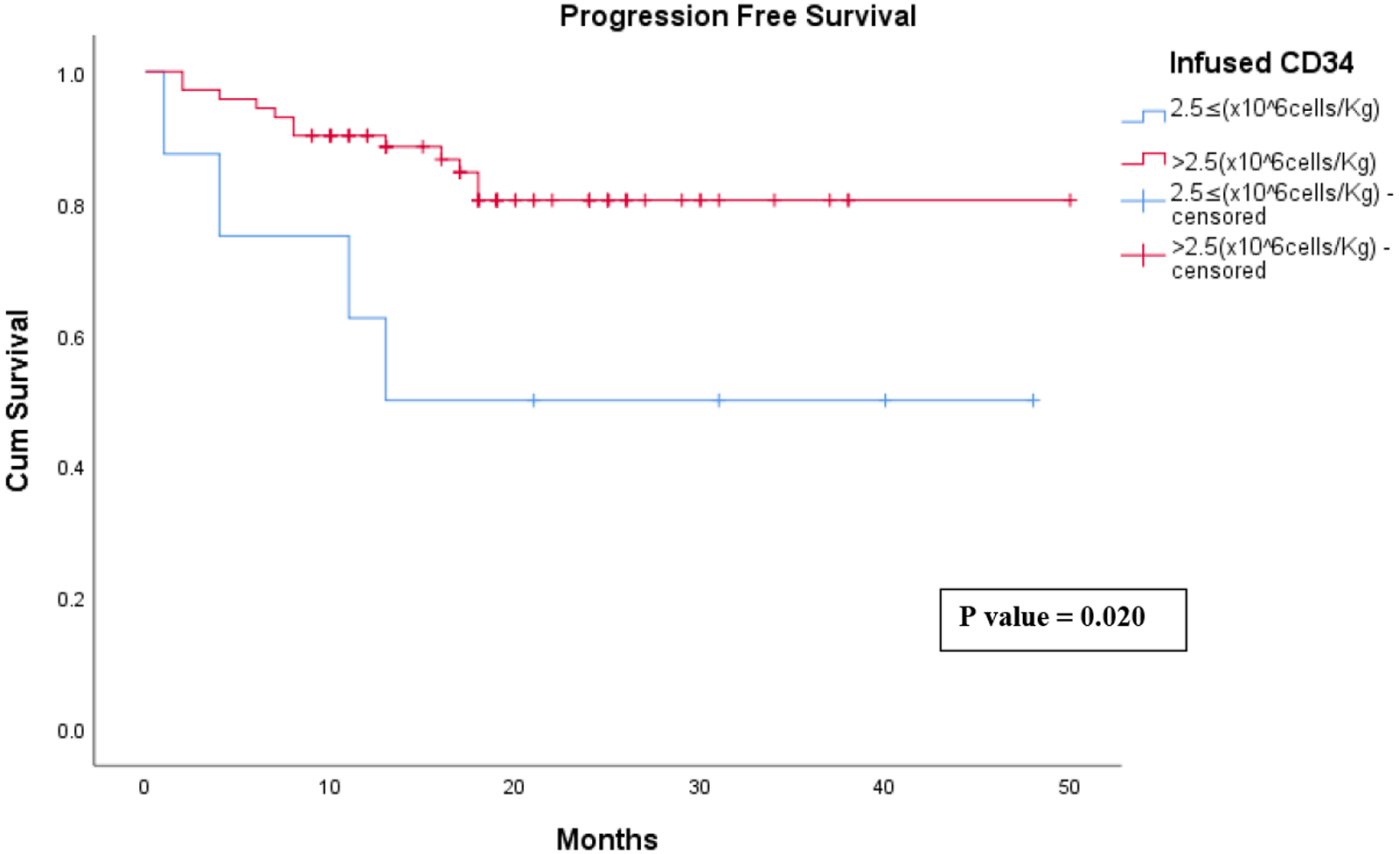
Progression free survival of patients infused CD 34 2.5≤ (×10^6^ cells/Kg) vs. >2.5 (×10^6^ cells/Kg).

Patients infused with a CD34 count >** **2.5 (×10^6^ cells/Kg) had a faster platelet engraftment by more than 2 days compared to the patients infused with a CD34 count 2.5 ≤ (×10^6^ cells/Kg) (*P* = 0.021). Moreover, patients infused with a CD34 count >** **2.5 (×10^6^ cells/Kg) had only a 17% relapse rate compared to 50% relapse rate among the patients infused with a CD34 count 2.5 ≤ (×10^6^ cells/Kg) (*P* = 0.025). Note the comparison of the progression-free survival (PFS) curves of the two groups where patients infused with a CD34 count >** **2.5 (×10^6^ cells/Kg) showed a better PFS rate over the first 50 months of follow up (*P* = 0.020) Figure 1, Table 4.

**Table 4:** Analysis of variables in association with outcomes.

Variable	Infused CD34+		
	2.5≤ (×10^6^cells/Kg)	>2.5 (×10^6^cells/Kg)	*P*-value
	(*N* = 8)	(*N* = 72)	
Length of Stay at Transplant in days *(mean)*	21.03	20.5	0.632
Days to ANC engraftment *(mean)*	11.4	11.1	0.637
Days to PLT engraftment *(mean)*	19.6	16.1	0.021
Progression/Relapse *N* (%)			0.025
Yes	4 (50%)	12 (17%)	
No	4 (50%)	60 (83%)	
Overall Survival Status *N* (%)			0.330
Dead	2 (25%)	9 (12%)	
Alive	6 (75%)	63 (88%)	
C. difficile infection *N* (%)			0.633
Yes	0 (0%)	2 (3%)	
No	8 (100%)	70 (97%)	
CLABSI^a^ *N* (%)			0.396
Yes	0 (0%)	6 (8%)	
No	8 (100%)	66 (92%)	

^a^ CLABSI, central line associated blood stream infection.

## Discussion

This retrospective single center study summarized the results of 84 patients with distinct clinical features comparing the efficacy of hematopoietic stem cell mobilization regimens in patients with various hematological malignancies, tried to figure out the predictive factors of poor mobilizers, and compared the survival rates of poor vs. good mobilizers. Based on the largest two studies performed studying the cutoff of peripheral CD34 cell dose to identify poor mobilizers by Bensinger et al. and Weaver et al., we have taken peripheral CD34 ≤ 2.5 × 10^6^ cells/Kg as an indicator of suboptimal number for an autologous transplantation (16, 17).

Usually patients with higher Body mass index (BMI) have less collected CD34 (18). However, this study observed the characteristics of patients with suboptimal collected CD34 ≤ 2.5 × 10^6^ cells/Kg vs. patients with optimal collected CD34 >** **2.5 × 10^6^ cells/Kg. It has been demonstrated that patients with more optimal collected CD34 cell count had a higher BMI average. Moreover, post-GCSF and plerixafor mobilization, patients with a more optimal CD34 cell count collection were at a statistically significant higher BMI than the patients with a suboptimal CD34 cell count (*P* = 0.024). On multivariate analysis higher BMI has shown more favorable peripheral CD34 cell count (OR = 1.215, *P* = 0.009) and better collected CD34 cell count post GCSF and plerixafor mobilization (OR = 1.254, *P* = 0.018). Based on this we can assume that higher BMI is a protective factor rather than a risk factor as it is has been considered by Donmez et al. (19). Healthy allogeneic donors with higher BMI have greater CD34 collected because this is compared to the weight of the recipient, especially if the recipient's weight is less than that of the donor; however, for autologous donors with higher BMI those generally will have lower yield. Chen et al. also considers that BMI positively affects the peripheral blood progenitor cell yield in healthy donors before and after mobilization with GCSF (20). In addition, a huge retrospective study with a sample of 2,503 allogenic transplant patients done by the Fred Hutchinson Cancer Research Center, concluded that underweight patients (BMI ≤ 18.5) and very obese patients (BMI ≥ 35) were both poor mobilizers (21). Taking into consideration our sample of patients, most of our patients fall into the categories lying in between the previously mentioned BMIs. This makes that our assumption is valid for BMIs ranging between 18.5 and 35. On the other hand, there has been no studies done yet to identify the cut off after which higher BMI becomes a risk factor rather than a protective one according to Gruppo Italiano Trapianto Midollo Osseo (GITMO) consensus (22).

MM patients have shown a more favorable CD34 cell count than Lymphoma patients (OR = 4.349, *P* = 0.027) however this favoritism was to a lesser extent after mobilization with GCSF and plerixafor. Donmez et al. and Sancho et al. also have considered Lymphoma one of the most important risk factors for poor hematologic stem cell mobilization [OR (lymphoma/MM) = 19.92 and *P* = 0.092] (19, 23).

Patients with bone marrow involvement were also noted to have suboptimal CD34 cell count than patients with unaffected bone marrow. On the other hand, we didn't see that bone marrow involvement had any effect on the CD34 cell count collected post G-CSF and plerixafor. However, as per Oliveri et al., bone marrow involvement is one of the minor criteria of predicted poor mobilizers according to the GITMO consensus, and disease infiltration ≥30% in the bone marrow is also considered to be an independent predictive factor for mobilization failure in predicted Poor Mobilizer (pPM) scoring (12, 22).

Finally our study further supported that infused CD34 cell count of 2.5 × 10^6^ cells/Kg is the closest estimate of the cutoff below which autologous transplantation is deemed to be suboptimal (24). The median time to platelet engraftment was approximately 16 days in patients infused with CD34 > 2.5(×10^6^ cells/Kg) which is less by more than 2 days compared to patients infused with CD34 ≤ 2.5 (×10^6^ cells/Kg) (*P* = 0.021). It has been proven in multiple studies that patients who received ≤2.5 (×10^6^ cells/Kg) CD34 cells had a significant delay in platelet engraftment when compared to patients who received CD34 > 2.5(×10^6^ cells/Kg) (*P* = 0.0001) (24–27). In addition, patients with the optimal cell count of >2.5(×10^6^ cells/Kg) had more than 30% improvement in progression-free survival (PFS) rate over 50 months compared to patients who received 2.5 ≤ (×10^6^ cells/Kg) CD34 cells (*P* = 0.02) which is comparable to other studies (28).

In conclusion, in this study we have confirmed that disease type (lymphoma) and lower BMI both pre-plerixafor and post GCSF and plerixafor are risk factors for poor mobilizers. We have seen as well that ≤2.5 (×10^6^ cells/Kg) collected and infused CD34 cells is associated with less PFS and longer platelet engraftment time. There are many other risk factors which are considered significant indicators of poor mobilizers and which can probably act as confounding variable in our case as well. These variables we either didn't find to be statistically significant, or we didn't study them at all. GITMO and pPM scoring consider failed previous mobilization, advanced age >65, prior extensive therapy, low pre-mobilization hemoglobin, previous mobilization failure and many others as significant risk factors of poor mobilizers. Those risk factors and others should be further tested in retrospective and prospective trials to demonstrate their effectiveness in identifying poor mobilizers ahead of time allowing to implement changes in those patients’ clinical management to avoid a very likely mobilization failure. Our study had several limitations. Taking into consideration the disease type like lymphoma as a risk factor for poor mobilization carries with it some limitations. Those limitations imply confounding variables like the intensity of chemotherapy regimen used, the number of lines of treatment and the duration of therapy received in lymphoma compared to other diseases like multiple myeloma where the transplant is done usually as first line and without intensive chemotherapy regimen before transplant.

Other confounding factors and unstudied variables that may have an influence on our results and conclusion are patients’ past medical history and comorbidities, patients' social history (smoking and alcohol consumption), medication history and many other variables.

To validate these promising results, prospective study cohorts of poor mobilizers adult patients undergoing ASCT are needed.

## Data Availability

The original contributions presented in the study are included in the article/Supplementary Material, further inquiries can be directed to the corresponding author.
